# Supplementary material for: A Metabolic Change towards Fermentation Drives Cancer Cachexia in Myotubes
Source: Biomedicines. 2021 Jun 20;9(6):698. doi: 10.3390/biomedicines9060698 (PMC8234377; doi:10.3390/biomedicines9060698)
Supplement: Supplementary file 1 [file biomedicines-09-00698-s001.zip › biomedicines-1256148-supplementary.pdf]

Supplementary figure S1

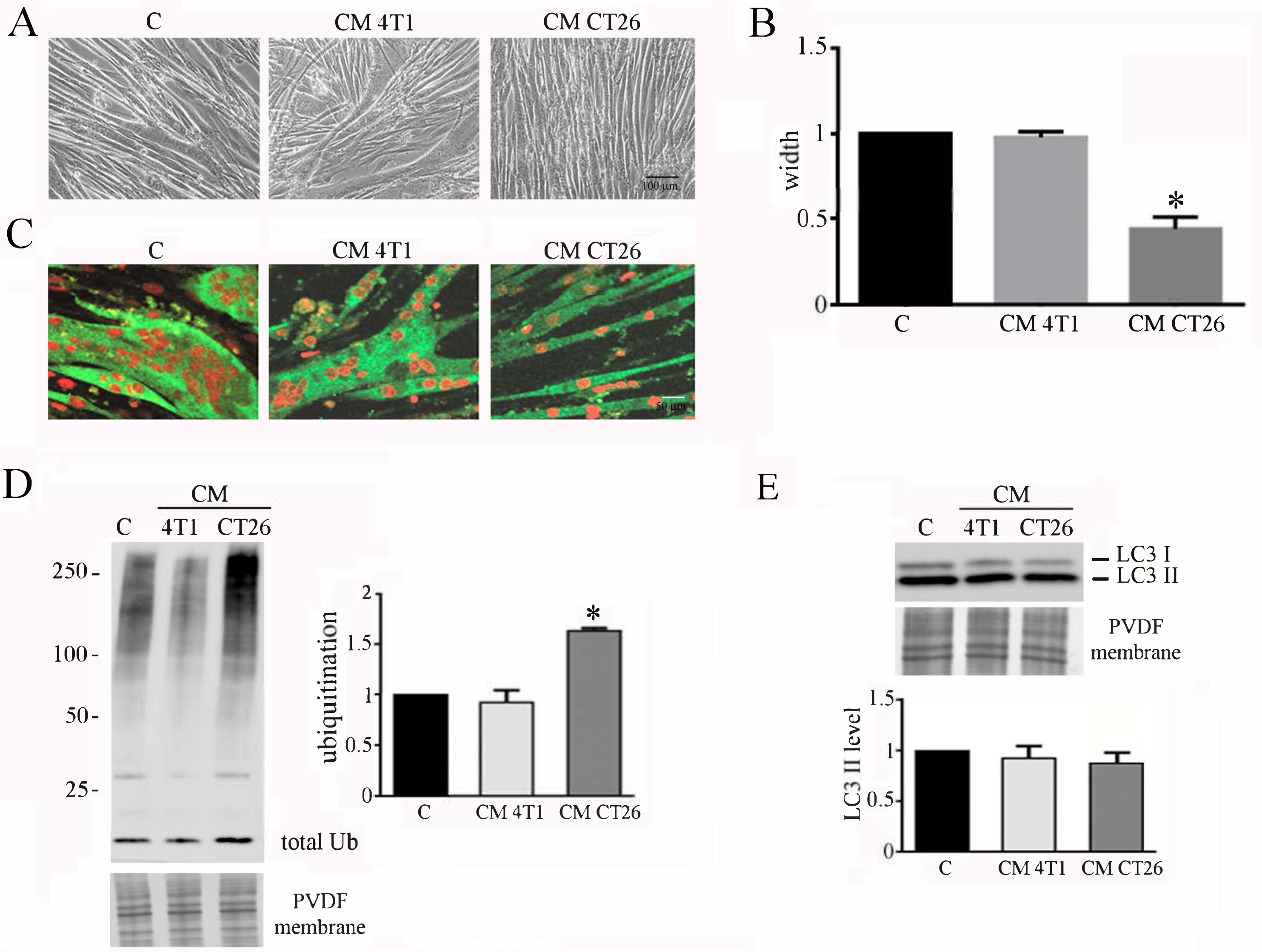

<u>CM CT26 induces cachexia in myotubes.</u> C2C12 myoblasts has been differentiated for four days and then treated with CM 4T1 or CM CT26 or differentiation medium for 24 hours. A) Representative images by optic microscope and C) by confocal analysis of myotubes after 24 hours of treatment. In C), green staining shows Myosin Heavy Chain (MHC) while Propidium Iodide (PI) indicates nuclei (in red). B) Analysis of myotube width. Myotube size has been measured using Image J, in at least ten random fields, and reported in the bar graph. D) Ubiquitination and E) LC3 levels in treated myotubes by immunoblot. Bar graphs show total ubiquitination and LC3 II level calculated using Coomassie stained PVDF membrane for the normalization. The values in the bar graphs are reported as folding increase considering control myotubes (indicated with C) as 1. n = 4. *$p<0.05$.

Supplementary figure S2

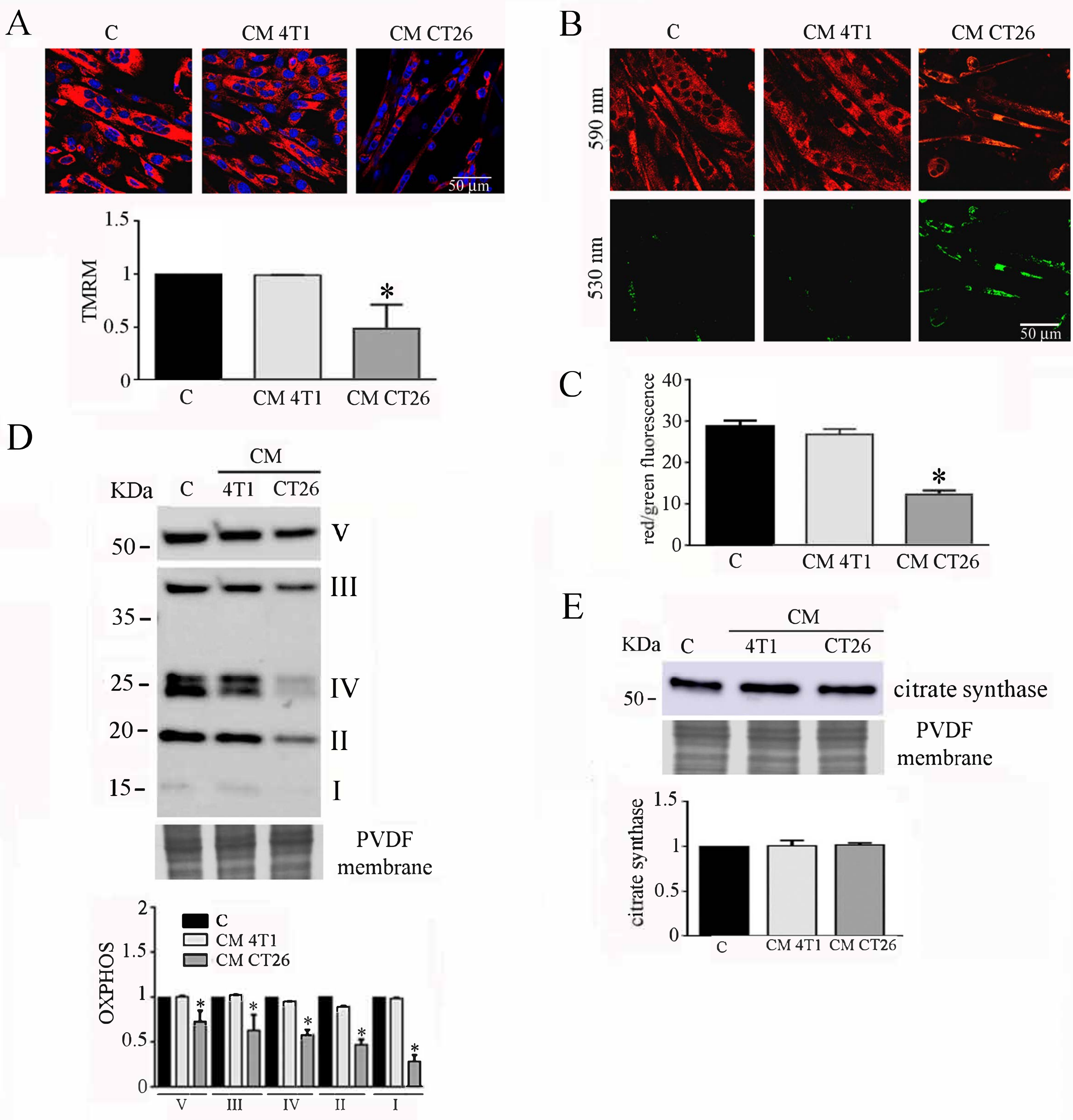

Analysis of mitochondria in myotubes treated with CMs. Four days differentiated myotubes were treated with CM 4T1 or CM CT26 or differentiating medium (C, control) for 24 hours. A) Analysis of mitochondria membrane potential by the fluorescent probe TMRM (1 µM final). B) Analysis of mitochondrial potential using JC-1 probe (5 µM final). Both TMRM and JC-1 probes have been added to CM-treated myotubes for 15 minutes after 24 hours of treatment and then immediately observed under confocal microscope. A) and B) panels show representative images of three independent experiments having similar results. C) Ratio between red and green fluorescence due to JC-1 probe. D) Immunoblot analysis of mitochondrial complexes (OXPHOS) and E) Immunoblot analysis of citrate synthase. Coomassie stained PVDF membranes were used for normalization. Bar graphs show citrate synthase and OXPHOS expression level. All the values in the bar graphs have reported as fold increase considering control myotubes as 1. C, control myotubes. n= 4; *$p<0.05$.

Supplementary figure S3

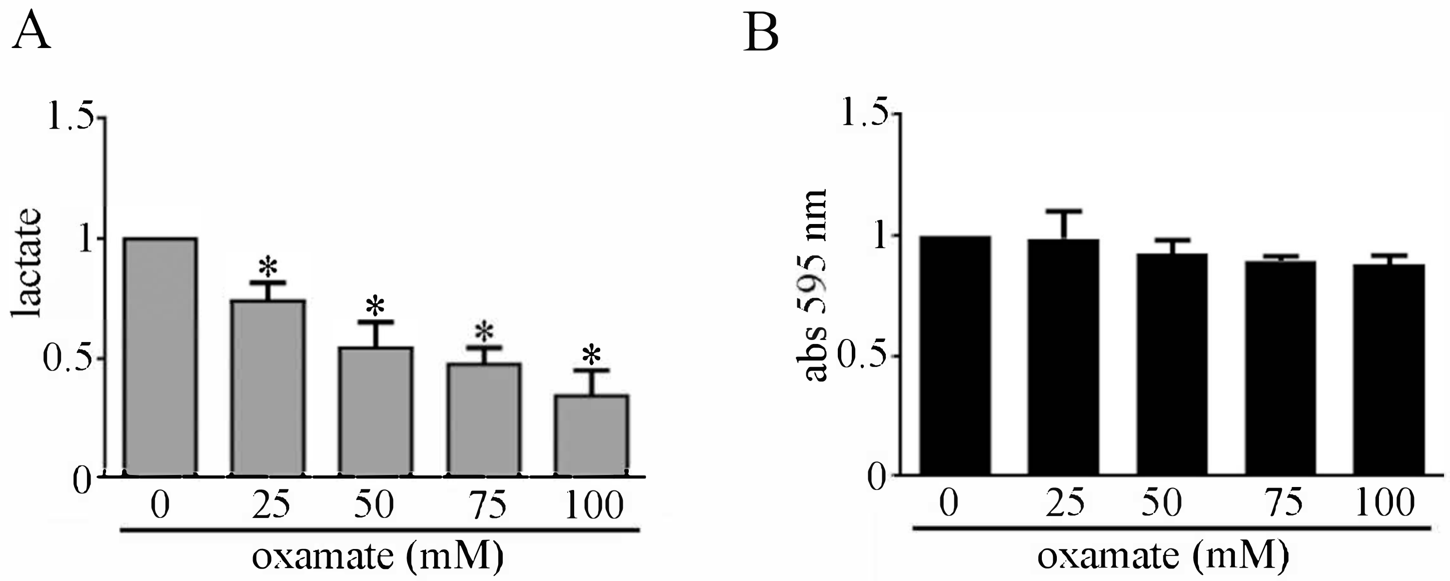

Four day differentiated myotubes were treated with increasing concentration of oxamate for 24 hours. A) Analysis of lactate amount in the medium. B) Analysis by MTT assay of myotube viability after treatment with different concentration of oxamate. All the values in the bar graphs have been reported as fold increase considering control myotubes as 1. n = 3. *p<0.05

Supplementary figure S4

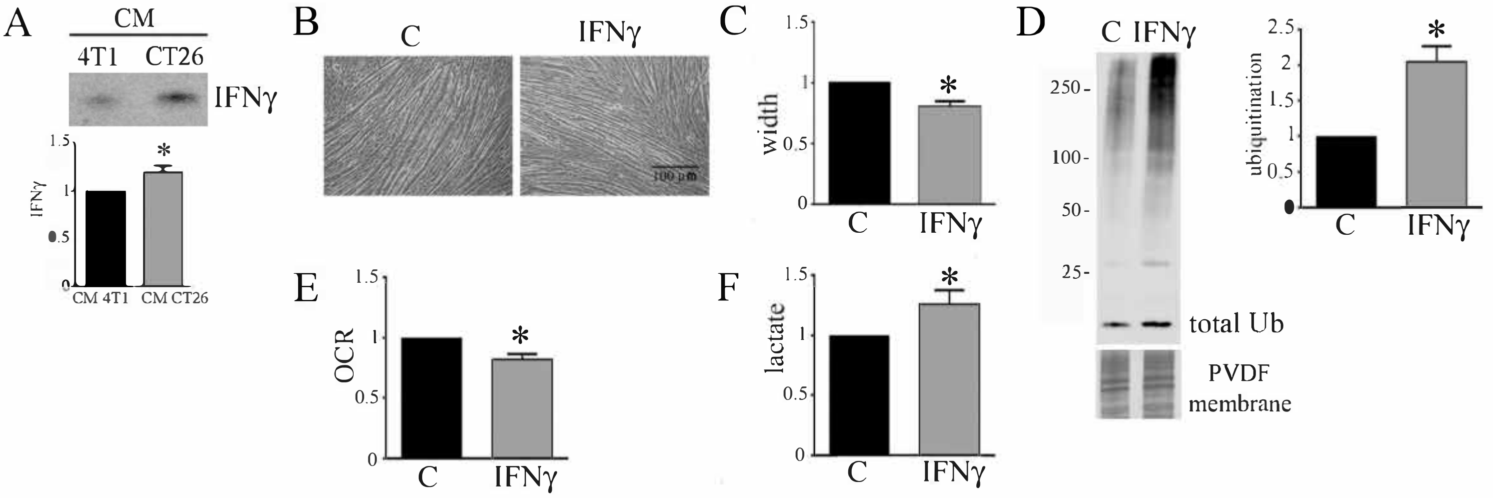

IFNγ induces a metabolic shift in myotubes associated with cachexia. A) Anti-IFNγ immunoblot performed in CM 4T1 and CM CT26. IFNγ level has been normalized on CM protein content. Four day differentiated myotubes are treated with IFNγ (120 ng/ml) for 24 hours, except untreated myotubes (indicated as C) that are maintained in differentiating medium for the same period. After the treatment, B) Phenotypic effect, observed by optic microscope, C) Measure of myotube width, D) Anti-ubiquitin immunoblot, E) Oxygen Consumption Rate (OCR) and F) lactate amount have been analysed. Normalized ubiquitination level was obtained using Coomassie stained PVDF membrane. OCR and lactate amount have been normalized on total protein content. All the values in the bar graphs have been reported as fold increase considering control myotubes as 1; n = 4; $*p<0.05$.

Supplementary figure S5

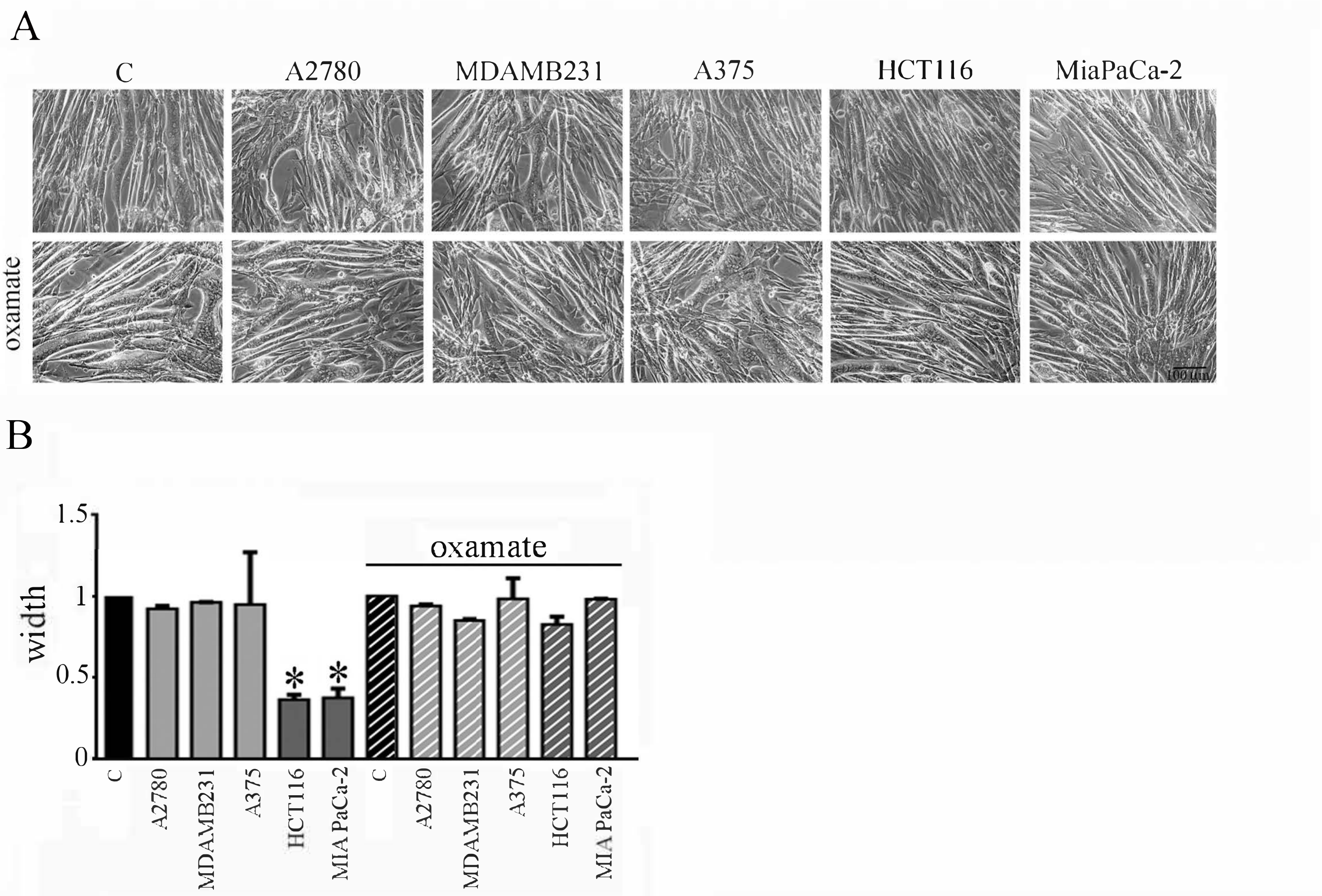

Representative images of myotubes treated with CMs from human cancer cell lines. Four day differentiated myotubes were treated for 24 hours with CM obtained from ovarian (A2780), breast (MDAMB231), melanoma (A375), colon (HCT116) and pancreatic (MiaPaCa-2) human carcinoma cells. B) Myotubes width after the treatment with the different CMs. Untreated myotubes (indicated as C) were maintained in differentiating medium. Where indicated LDH inhibitor's oxamate (75 mM) were added to the media for 24 hours. All the values in the bar graphs have been reported as fold increase considering control myotubes (indicated with C) as 1. n = 4. *p<0.05
